# White Laser Realized via Synergic Second- and Third-Order Nonlinearities

**DOI:** 10.34133/2021/1539730

**Published:** 2021-03-23

**Authors:** Baoqin Chen, Lihong Hong, Chenyang Hu, Zhiyuan Li

**Affiliations:** ^1^School of Physics and Optoelectronics, South China University of Technology, Guangzhou 510641, China; ^2^Guangdong Jingqi Laser Technology Corporation Limited, Songshanhu, Dongguan 523808, China

## Abstract

White laser with balanced performance of broad bandwidth, high average and peak power, large pulse energy, high spatial and temporal coherence, controllable spectrum profile, and overall chroma are highly desirable in various fields of modern science. Here, for the first time, we report an innovative scheme of harnessing the synergic action of both the second-order nonlinearity (2^nd^-NL) and the third-order nonlinearity (3^rd^-NL) in a single chirped periodically poled lithium niobate (CPPLN) nonlinear photonic crystal driven by a high-peak-power near-infrared (NIR) (central wavelength~1400 nm, energy~100 *μ*J per pulse) femtosecond pump laser to produce visible to near infrared (vis-NIR, 400-900 nm) supercontinuum white laser. The CPPLN involves a series of reciprocal-lattice bands that can be exploited to support quasiphase matching for simultaneous broadband second- and third-harmonic generations (SHG and THG) with considerable conversion efficiency. Due to the remarkable 3^rd^-NL which is due to the high energy density of the pump, SHG and THG laser pulses will induce significant spectral broadening in them and eventually generate bright vis-NIR white laser with high conversion efficiency up to 30%. Moreover, the spectral profile and overall chroma of output white laser can be widely modulated by adjusting the pump laser intensity, wavelength, and polarization. Our work indicates that one can deeply engineer the synergic and collective action of 2^nd^-NL and 3^rd^-NL in nonlinear crystals to accomplish high peak power, ultrabroadband vis-NIR white laser and hopefully realize the even greater but much more challenging dream of ultraviolet-visible-infrared full-spectrum laser.

## 1. Introduction

In the past 60 years, the collective action of laser technology and nonlinear optics has continuously pushed the realm of lasers into an ever-increasing height and ever-expanding frontiers in terms of laser materials (gas, liquid, solid, semiconductor, and fiber), pulse duration (continuous wave, nanosecond, picosecond, femtosecond, and attosecond), spectral width, power, and energy. A laser machine, with deliberate infrastructure design of cavity, gain medium, and pump source, outputs one or several specific discrete wavelengths of continuous-wave coherent light or a series of phase-fixed optical pulses with high coherence but only covering a limited spectral bandwidth. When inputting these lasers into a nonlinear crystal or material, nonlinear optical interaction will convert part of the energy into new lasers of different discrete wavelengths or spectral bands. These processes can generate new lasers and expand the spectral band, while maintaining the high coherence of the input laser. Thus, with the assistance of nonlinear optical frequency conversion and expansion technologies, the coverage of the laser spectral bandwidth has reached an unprecedented level. Yet, it remains a grand challenge to construct an all-spectrum laser machine that outputs a laser beam covering an extremely broad spectral bandwidth comparable with our everyday sunlight, which ranges from ultraviolet to midinfrared (300-5000 nm), while at the same time exhibiting high spatial and temporal coherence (i.e., beam collimation and pulse compression capability far exceeding the incoherent solar blackbody radiation) as well as high power and energy.

An obvious way to realize this great optical science dream of an all-spectrum laser seems to be the well-established technique of optical supercontinuum generation (SCG), which has been widely applied in various fields of modern science disciplines, with applications in precise frequency metrology [1–3], pulse compression [4], and optical coherence tomography [5]. In the past decades, many significant works on broadband SCG have been reported in systems including microstructure optical fiber [6–10], waveguide [11–16], and bulks [17–19]. The most popular routine towards SCG is harnessing the third-order optical nonlinear effect against a high peak power pump pulse of femtosecond or picosecond laser, including four-wave mixing, self-phase modulation (SPM), stimulated Raman scattering, in silica, fluoride, chalcogenide glass, and other noncrystalline materials. These materials have significant third-order nonlinearity (3^rd^-NL) coefficient *χ*^(3)^ but zero second-order nonlinearity (2^nd^-NL) coefficient *χ*^(2)^ due to the existence of inversion-symmetry in atomic structure of material. Appropriate dispersion design and control to have zero group velocity dispersion point or wavelength are requested to facilitate phase matching and distortion-free pump pulse transportation. Besides, the power density should be very high up to tens of GW/cm^2^ in order to ignite significant 3^rd^-NL interaction and considerable energy conversion efficiency from pump pulse to SCG pulse. One popular way is to concentrate the pump laser pulse energy at a tiny space region such as the core of a microstructure fiber [6–10], but this limits the pulse energy to the nanojoule level in order not to cause optical breakdown of fiber materials. Besides, due to imperfect synthesis and preparation of these microstructure fibers, the special and temporal coherence of input pump laser will not 100% pass on and copy into the signal laser, leading to some certain degradation of coherence of the output SCG. An alternative way is to use a very high energy pump pulse in bulk materials with the optical energy diluted in a large cross-section of the laser beam [17–19]. However, the dispersion of bulk material is hard to engineer to an ideal level so that the expansion of the SCG bandwidth is well below the scheme of the microstructure fiber. In short, due to various material, structural, and dispersion engineering limitations, this 3^rd^-NL scheme is so far still far away from accomplishing the dream of an all-spectrum laser.

Because *χ*^(2)^ is generally 5-6 orders of magnitude larger than *χ*^(3)^, it is much easier to have significant frequency conversion using 2^nd^-NL than 3^rd^-NL effects. Perhaps for this reason, in the long history of nonlinear optics advancing laser technology, it was various 2^nd^-NL optical effects, including notably the second-harmonic generation (SHG), sum-frequency generation (SFG), differential-frequency generation, parametric oscillation, and amplification processes, that were dominantly adopted to achieve laser frequency conversion for various continuous wave, nanosecond, picosecond, and femtosecond laser systems with low, modest, and high energy levels. Another reason is that various high-purity and broadband-transparency nonlinear crystals, including lithium niobate (LN), beta-BaB_2_O_4_ (BBO), lithium triborate (LBO), and KBe_2_BO_3_F_2_ (KBBF), that possess large *χ*^(2)^ have become mature after long time exploration and development, so that the coherence of the pump laser has been maintained to a high extent after passing these crystals. In these nonlinear crystal materials, the phase matching between the pump and output signal laser beams is the key to maintain high-efficiency frequency conversion. For this purpose, the birefringence phase matching (BPM) scheme [20–22] using the natural birefringence of nonlinear crystals and the quasiphase matching (QPM) scheme [23, 24] by using, for example, the periodic poled lithium niobate (PPLN), have been dominantly used. Nonetheless, these two schemes each have advantages and disadvantages in performance. One of the most serious problems with both BPM in nonlinear crystals and QPM in PPLN is their severe limitation in working bandwidth, namely, the spectral bandwidth where sufficiently high conversion efficiency between pump and signal laser via the nonlinear crystal is quite narrow, usually in the level of several nanometers. As a consequence, it is difficult to use the 2^nd^-NL scheme for realizing SCG, since SCG necessarily involves nonlinear optical interactions with very broad bandwidth. Perhaps for this reason, the discussion upon this scheme was relatively rare in the past literatures. But one thing is clear; this scheme in its standard formula is also very far from accomplishing the dream of the all-spectrum laser.

Then, how about the scheme combining 2^nd^-NL and 3^rd^-NL in a monolithic single nonlinear optical material platform? In this work, we will address this fundamental issue by showing and discussing the physical principle, the material design and fabrication, optical characterization, and practical performance of a successful visible to near-infrared (vis-NIR) white laser realized by a chirped LN nonlinear photonic crystal (NPC) that involves the synergic action of 2^nd^-NL and 3^rd^-NL simultaneously under the pump of a NIR femtosecond laser which is centered around 1200-1500 nm and has a modest bandwidth of 100 nm.

## 2. Principle of Synergic Action of Second- and Third-Order Nonlinearities

**Figure 1:**
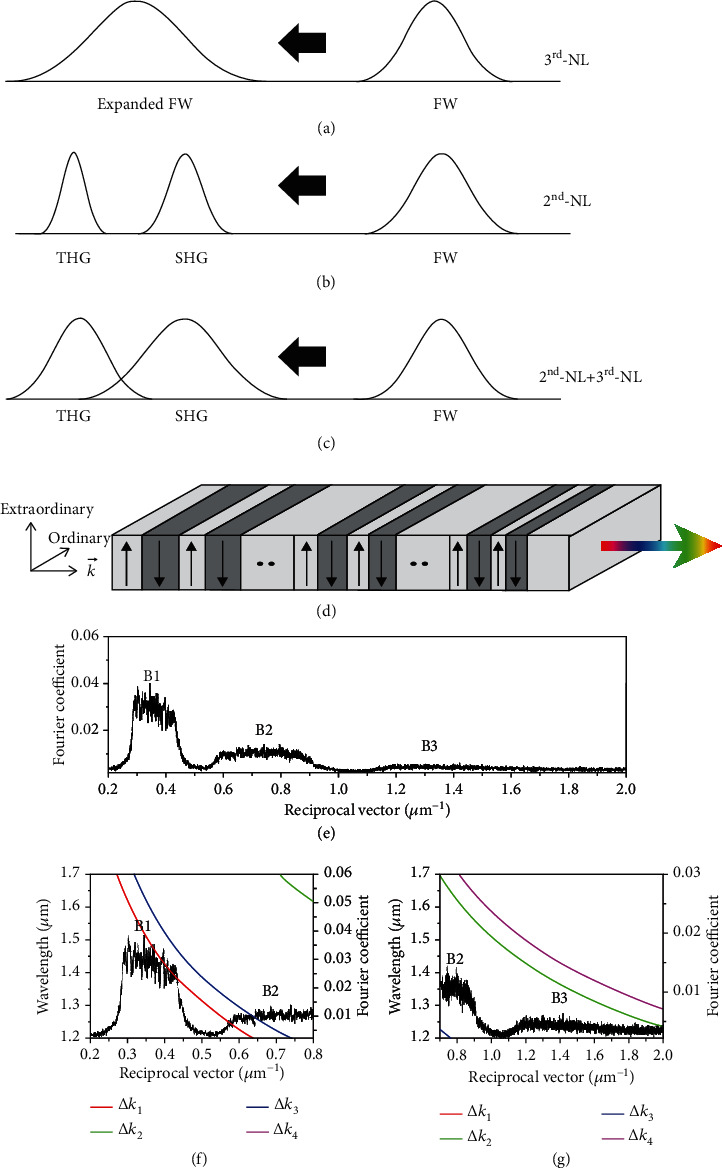
Principle for second- and third-order nonlinearities (2^nd^- and 3^rd^-NL) for generating vis-NIR white laser via CPPLN nonlinear crystal. (a) Bandwidth expansion of femtosecond lasers via 3^rd^-NL processes. (b) Simultaneous broadband SHG and THG via cascaded 2^nd^-NL QPM upconversion upon near-infrared pump femtosecond laser in CPPLN. (c) Synergic action of 2^nd^- and 3^rd^-NL for generating SHG and THG with much broadened bandwidth, leading to white laser with spectrum covering the whole visible band. (d) Schematic diagram of the structural geometry of a CPPLN structure. The up or down arrows indicate positive or negative domains, respectively, which enables upconversion of near-IR femtosecond into white laser. Under the pump of NIR femtosecond laser with arbitrary polarization, the CPPLN crystal can generate vis-NIR white laser. (e) Calculated distributions of the effective nonlinear coefficient as a function of the RLV for designed CPPLN sample. (f, g) Combined plots of the phase-mismatch curves for various three-wave mixing processes in a homogeneous LN crystal and the effective nonlinear coefficient curve for CPPLN structure in SHG and THG processes.

The condition to realize white laser covering the entire visible band and around is easy to understand and describe in principle, although in practice, it is not easy to satisfy: Have an input pump laser with a bandwidth as large as possible and a nonlinear material having an operation bandwidth as large as possible to upconvert the pump laser into a white laser. The basic physics principle for synergic action of second- and third-order nonlinearities, which will be taken in this work, is briefly illustrated in Figures 1(a)–1(c). As depicted in Figure 1(a), a femtosecond pulse laser (FW) with sufficiently high peak power and intensity (but not high enough to induce optical damage) passing through a LN nonlinear crystal would naturally ignite various 3^rd^-NL processes, with self-phase modulation being the dominant one for the LN crystal material, generating new spectral components causing considerable broadening of the spectral bandwidth of the input FW pump laser. The magnitude of spectral broadening depends on the pulse power density and the crystal sample length (generally ~1 cm in our work). On the other hand, as depicted in Figure 1(b), the FW femtosecond laser pulse would also ignite various 2^nd^-NL processes, where the cascaded SHG and THG (via SFG of FW and SHW) would be the dominant ones provided that the condition of ultrabroadband QPM could be satisfied, leading to creation of SHG and THG laser pulses transporting along with the FW pulse. When the conversion efficiency is high enough, these SHG and THG pulses still have high enough intensity to ignite 3^rd^-NL effects like SPM to further broaden their own spectral bandwidth. Then, as depicted in Figure 1(c), both 2^nd^-NL and 3^rd^-NL would act simultaneously in a practical sample, and their synergic operation would lead to creation of SHG and THG laser pulses with much broadened spectra compared with the situation when 2^nd^-NL and 3^rd^-NL act separately. The final consequence of this synergic action of 2^nd^-NL and 3^rd^-NL is the output of an ultrabroadband pulse white laser.

Let us first handle the critical problem of 2^nd^-NL upconversion bandwidth of LN nonlinear crystal. In a classical PPLN QPM scheme, one modulates the distribution of *χ*^(2)^(*z*) along the laser beam propagation direction with +*χ*^(2)^ and −*χ*^(2)^ arranged consecutively and periodically. The consequent NPC exhibits a series of reciprocal lattice vectors (RLV) *χ*^(2)^(*G*), which are called RLV lines (or equivalently QPM lines) because of the very narrow bandwidth. They are explored to counter the LN material dispersion induced phase mismatching for a specific nonlinear process via the QPM mechanism. With the maturity of manufacturing technology [25–29], various fascinating works have been done based on the QPM scheme [30–33]. However, despite the great flexibility, QPM in PPLN and other NPC structures suffers from a very small operation bandwidth. Thus, it is believed that the QPM scheme is more suitable for the frequency conversion of narrow-band continuous wave and nanosecond and picosecond laser rather than for a broadband femtosecond laser, not to say the extremely broadband SCG process.

Fortunately, things become different when one goes beyond the scheme of PPLN. In a previous work [34], we have shown that a single chirped PPLN (called CPPLN) crystal, which is made by introducing a chirped modulation upon the original periodic function of *χ*^(2)^(*z*) so that the series of RLV lines evolve into a series of RLV bands (or QPM bands), might be the solution. These bands can offer all the QPM bands that are requested by a number of cascaded three-wave mixing (SHG and SFG) upconversion 2^nd^-NL processes. As a result, simultaneous 2-8 high harmonic generation (HHG) and 350-1000 nm vis-NIR SCG (corresponding to 5-8 HHG) are created from a single CPPLN crystal under the pump of midinfrared (centered at ~3.6 *μ*m) femtosecond pulse laser. Although the conversion efficiency is as high as ~18%, the power of SCG is limited to a few mW because the pump power of the mid-IR femtosecond laser is only about 20 mW (with a repetition rate of 1 kHz, energy per pulse of 20 *μ*J, and pulse duration of ~50 fs) due to the technical limitation of the current mid-IR laser industry. To enhance the peak power and pulse energy level of the output white laser, it is highly desirable to greatly increase the pump power level of the FW pulse laser and further enhance the conversion efficiency simultaneously. For this reason, in this work, we turn to use NIR femtosecond laser with an energy per pulse of ~100 *μ*J level as the FW pump source. Besides, we make a specific design of CPPLN to match this pump condition, adopting low-order RLV bands with larger effective nonlinear coefficient *χ*_eff_^(2)^ to maintain broadband QPM conditions and enhance 2^nd^-NL interactions and energy conversion.

## 3. Sample Design and QPM Analysis of CPPLN

In the traditional PPLN scheme for QPM, the effective wave vector mismatching is described by Δ*k*_eff_ = Δ*k*_0_ − *G*_*m*_, where the extra RLV provided by the PPLN structure is *G*_*m*_ = 2*mπ*/*Λ*, with *Λ* being the poling period of the PPLN sample and *m* representing the QPM order. One can easily choose appropriate values of *Λ*, *m*, and *G*_*m*_ so that Δ*k*_eff_ = Δ*k*_0_ − *G*_*m*_ = 0, i.e., phase matching is satisfied. The poling period consists of a positive domain *l*_+_ and a negative domain *l*_−_. A PPLN structure with the fixed period can only provide a series of discrete RLV and QPM line, and the symmetric positive and negative domains will suppress the even order QPM, which is unfavorable for broadband nonlinear upconversion processes.

In contrast, the CPPLN nonlinear crystal as depicted in Figure 1(d) has a modulated period varying along the propagation direction. The continuously varied periods can provide a series of broad RLV bands to compensate the phase mismatching of the multiple broadband upconversion processes. The poling period of CPPLN crystal is designed to follow the formula *Λ*(*z*) = *Λ*_0_/[1 + (*D*_*g*_*Λ*_0_*z*/2*π*)] along the propagation direction (denoted as +*z*), where *Λ*_0_ is the initial period length and *D*_*g*_ is the chirp rate. Here, the central period is deliberately designed to achieve the first-order QPM for SHG at the NIR wavelength of 1.45 *μ*m, and an optimized CPPLN structure with chirp rate *D*_*g*_ = 14.2 *μ*m^−2^ is chosen to provide three RLV bands suitable for the broadband SHG and THG (via the cascaded SHG and SFG processes) against the pump NIR femtosecond pulse laser. The designed lattice pitch size reduces from 22 to 14.27 *μ*m, and the total length of the CPPLN sample is about *L* = 11 mm. The size asymmetry between positive and negative domains in the CPPLN supports both the odd and even order QPM mechanisms and provides more broadband RLV bands to match the broadband tunable feature and supercontinuum output. To ensure broadband SHG and THG simultaneously, the chirped poling period is obtained by varying the widths of the positive and negative domains simultaneously, but the negative domain is always 3 *μ*m longer than the positive part in one period.

To clearly illustrate the nature of physics for vis-NIR white laser, the effective nonlinear coefficient model is adopted to make qualitative analysis of designed CPPLN sample in terms of the availability of QPM and energy conversion efficiency [35, 36]. The quantitative distribution of the designed RLV bands *χ*^(2)^(*G*) can be calculated by Fourier transform to the domain-structure position function, *χ*^(2)^(*z*), which takes a value of +*χ*^(2)^ for positive domains and −*χ*^(2)^ for negative domains, with *χ*^(2)^ being the nonlinear permittivity for a bulk LN crystal. The new quantity *χ*^(2)^(*G*) is called the effective nonlinear coefficient *χ*_eff_^(2)^ and serves the same role of the usual nonlinear permittivity for a homogeneous nonlinear crystal in this CPPLN [35, 36]. Basically, a larger *χ*^(2)^(*G*) means a stronger 2^nd^-NL effect and thus a higher QPM conversion efficiency, although their correlation will not be a simple linearity. The calculated *χ*^(2)^(*G*) spectrum of the CPPLN sample, as plotted in Figure 1(e), exhibits three continuous RLV bands with balanced bandwidth and considerable Fourier coefficients, which are located in [0.27, 0.45] for band B1, [0.55, 1] for band B2, and [1.1, 2] for band B3, all in units of *μ*m^−1^. To fully illustrate the mechanism of QPM nonlinear processes in the designed CPPLN, we plot the phase mismatching curves for both SHG and THG versus the FW wavelength alongside the RLV spectrum in Figures 1(f) and 1(g). The wave-vector mismatch Δ*k*_i_ for different nonlinear frequency upconversion processes that are responsible for SHG and THG in these figures are defined in Table 1. Two polarization states of the pump laser, the extraordinary light and ordinary light, are considered in terms of the *z*-cut LN slab. The phase mismatching curves intersect with the RLV bands throughout a broad wavelength range from 1.2 *μ*m to 1.6 *μ*m. It not only covers the spectral bandwidth of the FW pump pulse laser at a single center wavelength, but also covers the tunable range of these pump pulse lasers, which ranges from 1200 to 1500 nm.

Besides the geometric and physical parameters of the CPPLN and the pump laser power, another important factor influencing the conversion efficiency is the polarization state of the pump light. As the CPPLN is made in the *z*-cut LN thin-film single crystal (see Figure 1(d)), the largest nonlinear coefficient *d*_33_ of the LN material (about 27 pm/V) then becomes the element of 2^nd^-NL tensor participating in the three-wave mixing nonlinear interactions when the pump laser and the upconversion lasers are all extraordinary light (called as *e*-polarization for short). Obviously, this configuration is the most beneficial one to ignite efficient SHG and THG. At this polarization state, the Δ*k*_1_ and Δ*k*_2_ processes as defined and illustrated in Table 1 contribute to the QPM required by high-efficiency SHG and THG, respectively. As seen clearly in Figure 1(f), the desired wave-vector mismatching for the Δ*k*_1_ is completely covered by band B1 and partially covered by band B2, indicating that the SHG process is enabled by the CPPLN over the entire wavelength range of the pump source. According to Figures 1(f) and 1(g), Δ*k*_2_ is covered by bands B2 and B3, which is responsible for the THG process. Due to the smaller Fourier coefficient in B3 of long-RLV part, THG under the pump wavelength in the short-wavelength part may not be significant. In a word, the designed broadband RLV bands B1, B2, and B3 are beneficial for broadband SHG and THG simultaneously under a wide pump wavelength range.

On the other hand, when the pump laser and the upconversion lasers are in the ordinary light polarization state (see Figure 1(d) and Table 1 for details), as depicted in Figures 1(f) and 1(g), we find that bands B1 and B2 overlap with the wave-vector curve Δk_3_ responsible for SHG, and bands B2 and B3 both contribute to process Δk_4_ responsible for THG. As a result, in the ordinary light case, the SHG and THG with a broadband tunable feature can also be achieved simultaneously in the designed CPPLN sample. Because of the relatively smaller nonlinear coefficient (*d*_31_ = 5.45 pm/V) participating in the nonlinear optical interaction, the conversion efficiency of SHG and THG processes might not be as high as in the extraordinary light pump configuration, but it is still observable.

## 4. Results

### 4.1. Nonlinear Optical Experiments

**Figure 2:**
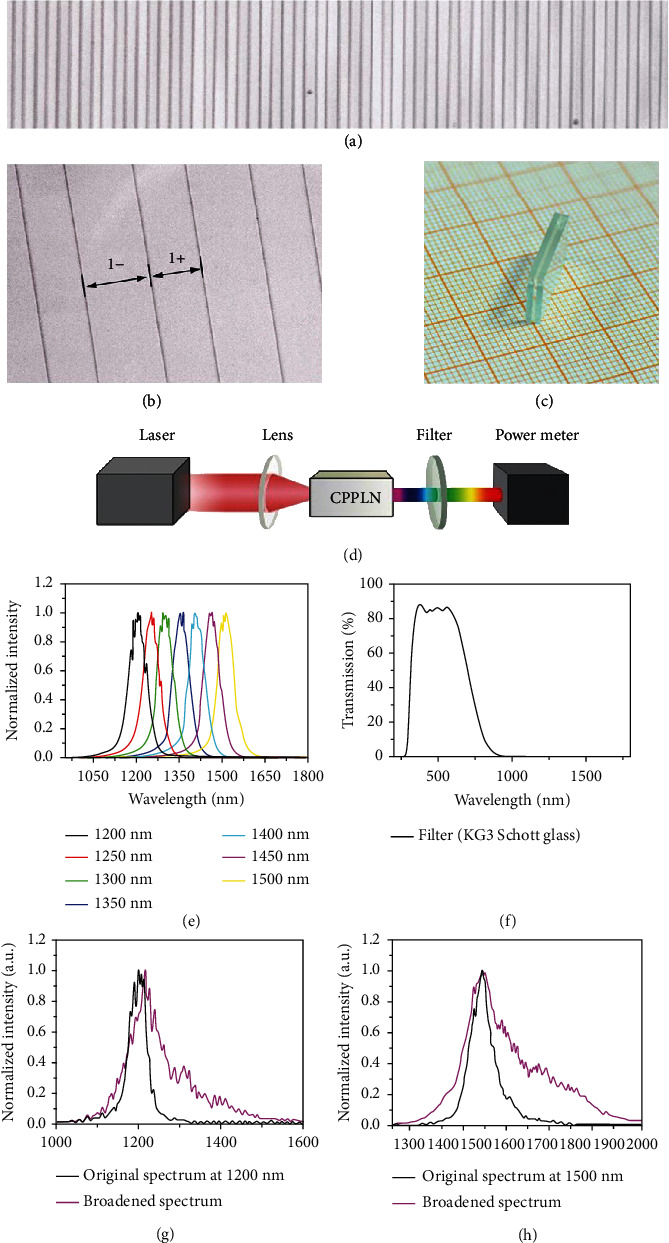
Experiment layout. (a) Microscopic image of the fabricated sample surface of a typical 1D CPPLN structure. (b) High-magnification view of the CPPLN sample, in which one can see that the lengths of negative and positive domains are unequal. (c) Fabricated CPPLN sample after encapsulation by sandwiching it within two glass sheets for convenience of experimental operating and testing. (d) Schematic diagram of the experimental setup. (e) Measured spectra of the tunable femtosecond pump source at each specific central wavelength from 1200 nm to 1500 nm of 50 nm intervals alongside the corresponding average power. (f) The transmission curve of filter. (g, h) Spectral broadening for the pump laser pulse with central wavelength 1200 nm and 1500 nm passing through a bare LN crystal.

The designed CPPLN samples are fabricated by the standard electric poling technique in a 0.5 mm thick *z*-cut LN planar slab, where the crystalline axis (also the optical axis) is perpendicular to the surface of LN slab [33, 34, 37]. For a typical sample, the microscopic details of positive and negative poled LN domains are explicitly illustrated in Figures 2(a) and 2(b), in which the asymmetric characteristic between negative and positive domains is clearly found as designed. The CPPLN sample has an overall size in length, width, and thickness of 11 mm × 6 mm × 0.5 mm, respectively. It is then sandwiched between two glass sheets to make appropriate encapsulation for convenience of nonlinear optical experiments (Figure 2(c)). The experimental setup is schematically illustrated in Figure 2(d). The input pump NIR femtosecond laser has a repetition rate of 1 kHz, a pulse duration of 50 fs, and an average power of 40-100 mW, corresponding to an energy level 40-100 *μ*J per pulse. The central wavelength of the NIR pump laser is set to continuously vary from 1200 to 1500 nm. The measured spectral profiles of the femtosecond pulse source are shown in Figure 2(e) for various central wavelengths. A filter with high transparency in the visible band while very low transparency above 1000 nm is used in the experiment to efficiently stop the pump laser to remove background noise and pass the signal white laser to determine its power level accurately. As the bandwidth of pump laser at each specific central wavelength is only ~100 nm, if only considering the 2^nd^-NL upconversion under SHG and THG, one would expect SHG and THG to at most occupy a bandwidth of 50 nm and 35 nm, respectively, in the visible band. Obviously, these two bands are well separated and are very far from covering the entire visible band. As a result, the dream of white laser would be completely dead in vain even when using this deliberate design of CPPLN.

However, things change dramatically when taking into account all involved 2^nd^-NL and 3^rd^-NL effects in the CPPLN. Considering a focus spot size of input laser beam around 1 mm, the peak intensity of the pump FW laser is around 50-120 GW/cm^2^. Here, the optical damage threshold for our CPPLN sample is estimated to be about 120 GW/cm^2^. Such a high level of laser field strength is sufficient to ignite remarkable 3^rd^-NL processes like SPM. Indeed, we have observed significant spectral broadening when the pump femtosecond laser passes through a bare LN crystal slab without action of QPM. Two examples are displayed in Figures 2(g) and 2(h) with the central wavelength located at 1200 and 1500 nm, respectively. The input femtosecond pulse laser has a bandwidth slightly below 100 nm, while the output femtosecond pulse laser has a greatly expanded bandwidth around 200-300 nm, exhibiting a two to three times spectral broadening. This spectral broadening is expected to also occur in the CPPLN crystal. When the pulse transports within the CPPLN, this 3^rd^-NL will work together with the 2^nd^-NL to make the upconversion SHG and THG have a much larger bandwidth than expected from pure 2^nd^-NL. Besides, we expect this pulse spectral broadening not only takes place for the pump laser but also takes place for the upconversion SHG and THG lasers, since they also have a pretty high level of peak power due to high upconversion efficiency. Then, the observed output white laser from the CPPLN sample should be a mixture contribution of these complicated 2^nd^-NL and 3^rd^-NL, which accords well with the physical picture of synergic action illustrated in Figures 1(a)–1(c).

### 4.2. White Laser Output with Controllable Chroma

**Figure 3:**
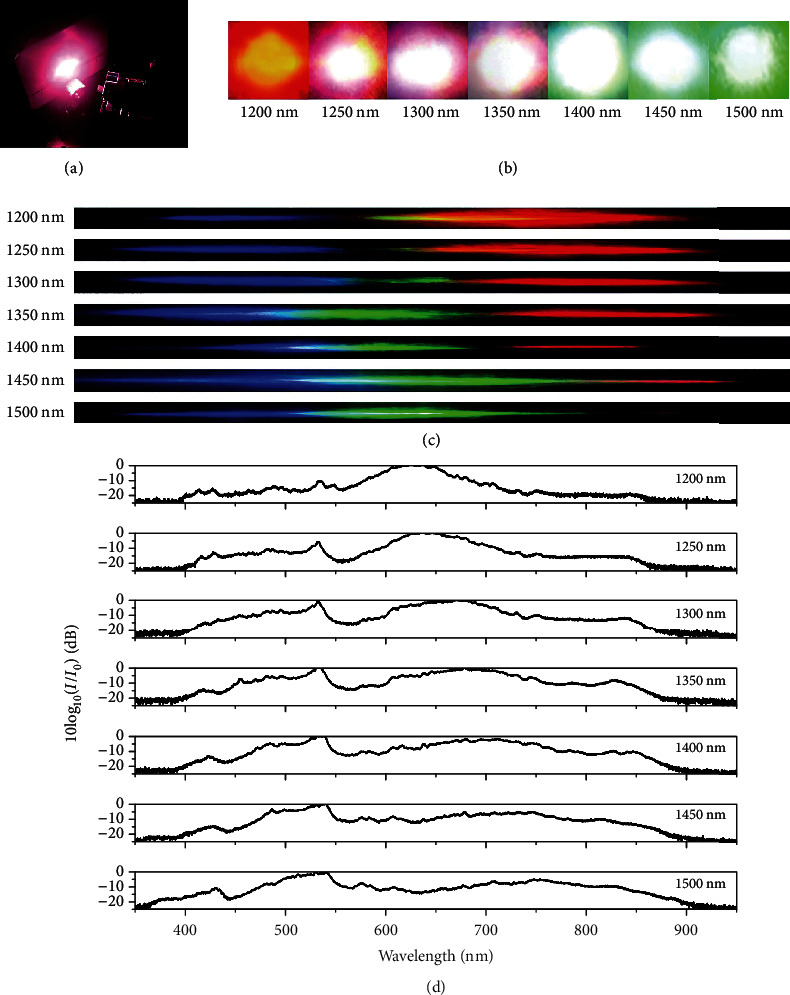
Upconversion supercontinuum generation when incident pump lasers are extraordinary lights. (a) Photograph of the sample setup with a dazzling output spot. (b) Photographs of upconversion spots. (c) The 1^st^-order diffraction beam and (d) supercontinuum spectra (normalized against the peak intensity) emitted from the CPPLN sample under the pump of femtosecond pulse laser with the central wavelength ranging from 1200 to 1500 nm. Here, the white laser under the pump of FW femtosecond laser with the central wavelengths 1200, 1250, 1300, 1350, 1400, 1450, and 1500 nm is found to involve a series of vis-NIR bands covering approximately 393-860 nm, 400-880 nm, 370-902 nm, 391-905 nm, 366-900 nm, 370-900 nm, and 360-905 nm, respectively, as estimated by a criterion of 20 dB. In short, the spectra of output white laser cover a series of vis-NIR bands ranging approximately from 400 to 900 nm in all cases of FW pump laser.

We evaluate the performance of the CPPLN samples by first measuring the upconversion process when the polarizations of pump lasers are extraordinary lights, where the electric field is parallel to the surface normal of the *z*-cut LN planar slab. In Figure 3(a), a dazzling spot is found immediately by sending a NIR pump laser beam through the CPPLN sample. The evolution of the output upconversion spots is recorded by camera and displayed in Figure 3(b). When the central wavelength of incident pump laser is continuously tuned from 1200 to 1500 nm with a 50 nm interval, the upconversion spot is observed with the eye-viewed colour changing from red to green, which clearly indicates that the overall chroma of the output laser is changing. To have a better idea about the frequency component of the output laser, we use a regular optical grating for a qualitative assessment against the output laser beam. As shown in Figure 3(c), the 1^st^-order diffraction beams involve continuous visible colour bands in all cases of pump laser, indicating that the upconversion lights are a supercontinuum white laser that covers the entire visible band. Besides, the intensity of various colour bands is not uniform, and the overall colour-band profile changes in different central wavelengths of pump laser.

An optical spectrometer is then used for accurate spectral analysis of the output white laser spots. From Figure 3(d), we can find that the spectra of output spots in all cases cover a series of vis-NIR bands ranging approximately from 400 to 900 nm, which is about 5 times the bandwidth of the input pump femtosecond pulse laser (slightly below 100 nm). The detailed structure of spectral profile in terms of various colour band intensities can be seen to correspond well with the picture of Figure 3(c). Owing to the small Fourier coefficient (and thus small *χ*_eff_^(2)^) in the long-RLV (short wavelength) part of band B3, THG becomes weak when the central wavelength of the pump is chosen at 1200 nm, and only SHG occurs significantly with the peak located at ~620 nm (first panel, Figure 3(d)). As the wavelength of the pump laser increases, larger Fourier coefficient comes into plays and makes contribution, so that THG becomes more and more significant, while at the same time, SHG maintains equal significance (second to sixth panels, Figure 3(d)). Besides, both SHG and THG occupy a bandwidth far larger than the 50 and 35 nm that are expected when only 2^nd^-NL plays roles.

To further quantify the upconversion efficiency, a bandpass filter is chosen and placed before the power meter to eliminate the residual pump energy. Considering the transmission performance of the filter, the calibrated average upconversion laser power can be calculated precisely. The detail of the experiment conditions and the estimated conversion efficiency are integrated and presented in Table 2. The conversion efficiency is all within 20-30%, which is quite a high value. The maximum value output power is ~19 mW at the 1300 nm pump laser with a power~70 mW, which is more than 6 times larger than the value (2.65 mW) obtained using the mid-IR power laser pump upon CPPLN, as reported in [34, 37]. Besides, we believe when antireflection coating at the front and rear interfaces of the CPPLN sample is used to eliminate the reflection and reduce the insertion loss, the upconversion efficiency will be even higher.

**Figure 4:**
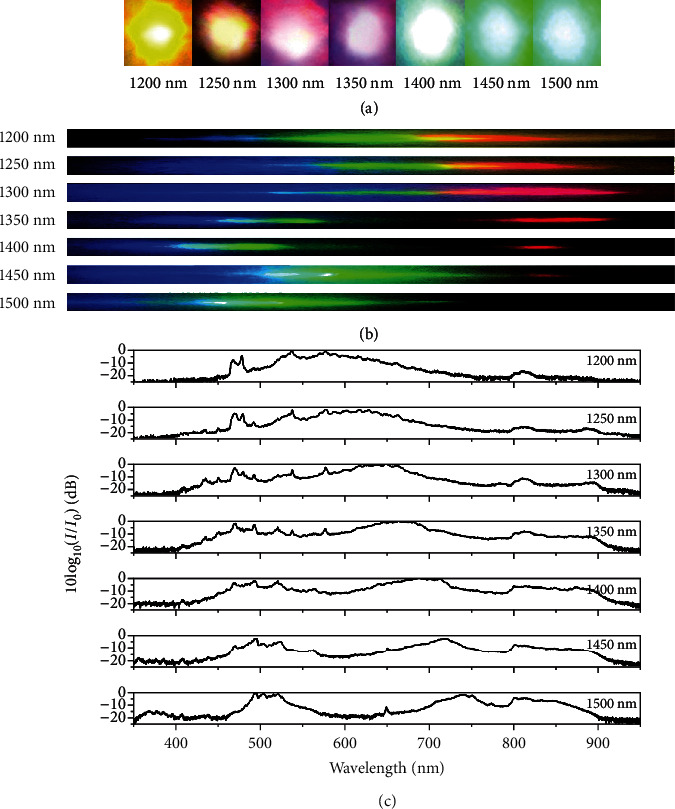
Upconversion supercontinuum generation when incident pump waves are ordinary lights. (a) Photographs of upconversion spot, (b) the corresponding 1^st^-order diffraction beam, and (c) normalized supercontinuum spectra emitted from the CPPLN sample at specific central wavelength of femtosecond pump source from 1200 to 1500 nm. Here, the white laser under the pump of FW femtosecond laser with the central wavelengths 1200, 1250, 1300, 1350, 1400, 1450, and 1500 nm is found to involve a series of vis-NIR bands covering approximately 397-860 nm, 400-888 nm, 360-905 nm, 350-910 nm, 350-900 nm, 360-900 nm, and 363-905 nm, respectively, as estimated by a criterion of 20 dB. In short, the spectra of output white laser cover a series of vis-NIR bands ranging approximately from 400 to 900 nm in all cases of FW pump laser. On the other hand, a notable feature in this case is the existence of a high efficiency band at 800-900 nm.

To see the effect of the pump laser polarization state, we rotate the CPPLN sample to ensure that the incident pump waves are ordinary lights. By varying the central wavelength of the femtosecond pump source from 1200 to 1500 nm with a 50 nm interval, we still find output of significant upconversion white laser in all cases. The upconversion spots and the corresponding spectra are illustrated in Figure 4, which indicate the successful implementation and modulation of white laser by using the designed CPPLN sample. From Figure 4(c), we find that the spectra of output spots in all cases also cover a series of vis-NIR bands ranging approximately from 400 to 900 nm, about 5 times the bandwidth of the input pump femtosecond pulse laser. It can be seen that the upconversion spectral profiles at this ordinary light pump is quite different from the case of extraordinary light pump, and this can be qualitatively understood from the QPM diagram as depicted in Figures 1(c) and 1(d) as well as Table 1. Note that the ordinary refractive index and extraordinary refractive index of the birefringent LN material exhibit slightly different features of dispersion. This causes the accurate condition to satisfy QPM to be somewhat different in these two pump laser polarization states and thus induces different output spectral profiles for pump laser at the same central wavelength from 1200 to 1500 nm with a bandwidth of about 100 nm.

A notable feature of Figure 4(c) is the existence of a high efficiency band at 800-900 nm, which is missing in Figure 3(c). We attribute this band to the optical parametric amplification between the high-frequency parts of the upconversion SHG supercontinuum and FW pump laser. The overall power and conversion efficiency at this ordinary light pump laser polarization is summarized in Table 3, which shows that the conversion efficiency is at the modest level of 5-8% and the power of output white laser has a maximum value of only 6.6 mW. Both quantities are much smaller than the extraordinary light pump case. The most important reason for this big contrast is that the ordinary light polarization state of pump laser only uses the *d*_31_ (~5.45 pm/V) nonlinear coefficient of LN, which is 5 times smaller than the *d*_33_ (~27.2 pm/V) nonlinear coefficient used in the extraordinary light pump laser polarization.

**Figure 5:**
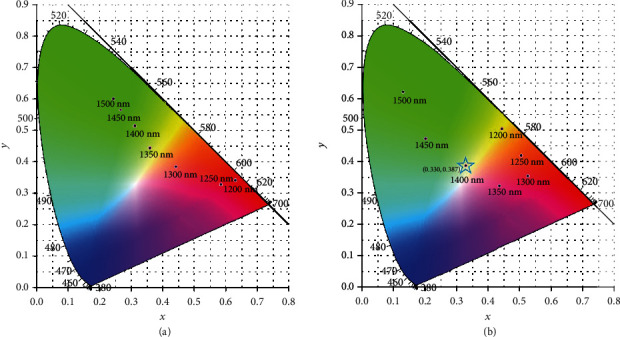
Chromaticity diagram. Chromaticity coordinate variation of the upconversion supercontinuum with respect to the central wavelength of near-IR pump laser with (a) extraordinary light and (b) ordinary light polarization states, respectively. The white emission colour is marked as the blue pentagram with its CIE coordinate.

Since the output visible-NIR white laser is versatile by pumping the CPPLN using 1200-1500 nm NIR femtosecond laser, it is interesting to further examine the overall visual colour of the white laser output from the designed CPPLN samples, which can be characterized by the quantity of chroma. The overall colours of the upconversion white laser are shown in the CIE chromaticity diagram (Figure 5) based on the measured optical spectra displayed in Figures 3(d) and 4(c) for the extraordinary and ordinary light polarizations of pump laser. The best performance in terms of chroma for white laser occurs at the condition of pump laser with 1400 nm central wavelength and ordinary light polarization, as explicitly denoted by the blue pentagram mark in Figure 5(b), where the CIE coordinate is (0.330, 0.387). For the extraordinary light polarization of pump laser, the best performance of chroma for white laser happens at the central wavelength 1350 nm, with the CIE coordinate being (0.350, 0.420).

## 5. Discussion

The above experiment data clearly indicate that vis-NIR white laser can be readily achieved through pumping a single ultracompact size CPPLN nonlinear crystal by NIR femtosecond pump lasers in a wide range of conditions of central wavelength, polarization, and intensity. The properties of white laser, including the intensity, spectra, and chroma, can be modulated in a broad range. This capability of CPPLN can be attributed to the synergic action of 2^nd^-NL and 3^rd^-NL that is enabled by the special QPM properties of CPPLN in combination with the high peak power and broad bandwidth nature of a pump femtosecond pulse laser. In more details, the following mechanisms play active roles. First, the 3^rd^-NL enabled by the peak power of pump laser will account for the initial spectral broadening effect, and new spectral components add into the pump laser pulse when it transports in CPPLN. Second, because of the sufficient broadband RLVs provided by the designed CPPLN sample, the 2^nd^-NL three-wave mixing upconversion processes, including direct SHG and indirect THG, against the original pump laser and all the 3^rd^-NL-induced new frequency components become very efficient in energy conversion. Third, the upconversion SHG and THG pulses will also encounter their own 3^rd^-NL effect and induce a new round of spectral broadening processes. All these three processes will intertwine with each and act collectively during the transport of the pump pulse laser in the nonlinear crystal. Consequently, although the original bandwidth of the pump laser is only about 100 nm in the near-IR region, the synergic action of these 2^nd^-NL and 3^rd^-NL effects can upconvert the energy of the high-peak power near-IR FW pump laser with a very high efficiency together with a significantly broadened spectra and result in supercontinuum vis-NIR white laser covering 400-900 nm. Moreover, because this synergic action of 2^nd^-NL and 3^rd^-NL subtly depends on pump laser conditions, it offers a promising route to control the properties of output vis-NIR white laser in terms of peak power, pulse energy, spectral bandwidth and profile, and overall chroma. This great flexibility is highly desirable for engineering the properties of vis-NIT white laser in application to ultrafast optics, display, information processing, spectral analysis, and other optical science and technology areas. In short, the most important innovation of this work is to present for the first time a route to explore and harness the synergic action of both 2nd-NL and 3rd-NL effects in a single nonlinear optical material to realize ultrabroadband white laser in the visible-NIR band and thus offer a novel and promising route to create white laser with high-peak power and high pulse energy.

Finally, although we have designed and fabricated a CPPLN nonlinear crystal to realize the ultrabroadband vis-NIR white laser with 19 mW in average power, 19 *μ*J in energy per pulse, high efficiency up to 30%, and an overall chroma close to perfect white laser, we still expect that these numbers can be greatly enhanced by adopting a femtosecond pulse laser with higher peak power and energy (e.g., increasing from 100 *μ*J to 1 mJ per pulse), narrower pulse duration (e.g., reduced from 50 fs to 25 fs), and larger bandwidth (e.g., increasing from 100 nm to 200-300 nm) as a pump source against CPPLN nonlinear crystals. Such stronger pump laser surely will greatly enhance both the 2^nd^-NL and 3^rd^-NL processes, as well as their synergic actions, leading to higher power and pulse energy, better spectral profile, larger bandwidth, and more perfect chroma of white light. Moreover, these experiences of vis-NIR white laser will shed new light to accomplish the dream of ultraviolet-visible-infrared all-spectrum lasers of high performance in terms of bandwidth, spectrum, energy, power, and coherence.

## 6. Materials and Methods

### 6.1. Fabrication of CPPLN Samples

We fabricate the CPPLN samples by using the electric poling technique at room temperature. Standard optical-grade *z*-cut LiNbO_3_ crystals of 0.5 mm thickness are used as the raw material. First, we deposit a thin layer of photoresist onto the +Z surface of the crystal and then lithographically pattern it with the designed chirped lattices. The exposed area is then contacted with liquid electrolyte which is made of saturated solution of lithium chloride in deionized water. The unexposed area is separated with the liquid electrolyte by the photoresist. Then, a pulse electric field of about 24 kV/mm is applied on two sides of the crystal to complete the domain reversion.

### 6.2. Experiment Setup of the Measurement

The pump pulse laser used to ignite SCG from the fabricated CPPLN sample comes from a Ti:sapphire femtosecond pulse laser (Coherent, Astrella USP, maximum pulse energy 6 mJ) together with an optical parametric amplification (OPA) frequency converter (Coherent, Topas, 240-20,000 nm). The repetition rate is 1 kHz and the duration is around 50 fs. The pump source is weakly focused by a *f* = 1000 mm fused silica lens and coupled into the polished end of the CPPLN sample. The power of the output laser is directly monitored by a regular power meter. To clarify the conversion efficiency of the upconversion laser, appropriate filters are carefully selected for nonlinear optical measurement setup, which should bandstop the pump laser and bandpass the upconversion laser.

## Figures and Tables

**Table 1:** Definition of the wave-vector mismatch Δ*k* as used in Figure 1 for different nonlinear three-wave mixing processes that contributes to SHG and THG.

Harmonic order	Wave-vector mismatching in three-wave mixing processes	QPM band
2^nd^ HG in *e*-polarization	Δ*k*_1_ = *k*_2_^*e*^ − 2*k*_1_^*e*^	B1, B2
3^rd^ HG in *e*-polarization	Δ*k*_2_ = *k*_3_^*e*^ − *k*_2_^*e*^ − *k*_1_^*e*^	B2, B3
2^nd^ HG in *o*-polarization	Δ*k*_3_ = *k*_2_^*o*^ − 2*k*_1_^*o*^	B1, B2
3^rd^ HG in *o*-polarization	Δ*k*_4_ = *k*_3_^*o*^ − *k*_2_^*o*^ − *k*_1_^*o*^	B2, B3

*k*_ij_ is the wave vector of the *i*th HG wave within a LiNbO_3_ crystal. The superscript *j* indicates the polarization of the mixing wave. The wave number is given by *k*_*i*_^*j*^ = *i* × *k*_0_ × *n*_*j*_(*λ*_*i*_), where *k*_0_ is the wave number of the FW in vacuum and *n*_*j*_(*λ*_*i*_) is the refractive index of LiNbO_3_ under the polarization of *j* at the *i*th HG wavelength *λ*_*i*_.

**Table 2:** The details of the experiments when the incident pump is composed of extraordinary lights.

Central wavelength of pump source	Average power of the pump source (mW)	Average power of upconversion supercontinuum in 400 to 900 nm (mW)	Upconversion efficiency
1200 nm	50	15.2	30.4%
1250 nm	67.6	18.25	27%
1300 nm	68.9	18.92	27.4%
1350 nm	66.2	17.8	26.9%
1400 nm	62.1	15.94	25.6%
1450 nm	50	13.2	26.4%
1500 nm	40.5	8.38	20.7%

**Table 3:** The details of the experiments when the incident pump is composed of ordinary lights.

Central wavelength of pump source	Average power of the pump source (mW)	Average power of upconversion supercontinuum in 400 to 900 nm (mW)	Upconversion efficiency
1200 nm	68.9	5.02	7.3%
1250 nm	90.5	6.64	7.3%
1300 nm	97.3	6.61	6.8%
1350 nm	85.1	4.97	5.8%
1400 nm	74.3	4.38	5.9%
1450 nm	67.5	4.29	6.3%
1500 nm	47.3	2.51	5.3%

## Data Availability

The data that support the finding of this study are available from the corresponding author upon reasonable request.
